# DRIVING RISK TOLERANCE AS A PREDICTOR OF OLDER ADULTS’ ABILITY TO DRIVE

**DOI:** 10.1093/geroni/igae098.2573

**Published:** 2024-12-31

**Authors:** Angela Curl

**Affiliations:** Miami University, Oxford, Ohio, United States

## Abstract

Driving is critical to mobility, autonomy, freedom of choice, and engagement for older adults, but many eventually stop driving. Evaluation of fitness to drive, risks related to driving, and personal preferences about driving all can play a role in driving cessation. Using 2022 data from the Health and Retirement Study (HRS; N=2,209), this study examined the relationship between driving risk tolerance and driving cessation for White and Black adults over age 65. Risk tolerance (key predictor) was measured as willingness to take risks while driving (0=“unwilling to take any risks” to 10=”fully prepared to take risks”). Self-reported ability to drive (yes/no) assessed driving cessation. Model 1 consisted of driving risk tolerance, controlling for education, gender, race, ethnicity and age, while Model 2 tested whether the relationship was mediated by self-rated eyesight, health, and memory (3 single-item measures, 0=poor to 4=excellent). Logistic regression results indicated that higher driving risk tolerance was associated with greater likelihood of ability to drive in Model 1 (OR=1.08, p=.01). Addition of health factors (Model 2) only weakly mediated this relationship, with driving risk tolerance having a slightly stronger impact on driving ability (OR=1.10, p<.01). Better eyesight (OR=1.76, p<.01) and health (OR=1.45, p<.01) were associated with greater likelihood self-reported ability to drive. As driving involves potential risks (e.g., traffic tickets, accidents), driving assessments should include perceptions of level of risk in discussions about driving cessation. Interventions designed to help older adults accurately assess their driving risk and how to minimize risks could promote driving ability/driving cessation.

